# Inflammatory changes in early dementia with Lewy bodies

**DOI:** 10.3389/fneur.2025.1640614

**Published:** 2025-08-26

**Authors:** Talene A. Yacoubian, Hongwei Qin, Amy Amara, Natividad Stover, Lauren Ruffrage, Yue Zhang, Richard Kennedy, David Geldmacher, Adam Gerstenecker, David G. Standaert, Etty N. Benveniste

**Affiliations:** ^1^Department of Neurology, Birmingham, AL, United States; ^2^Department of Cell, Developmental, and Integrative Biology, Birmingham, AL, United States; ^3^Department of Medicine, University of Alabama at Birmingham, Birmingham, AL, United States

**Keywords:** Parkinson’s disease, dementia with Lewy bodies, inflammation, cognition, neutrophils

## Abstract

**Objective:**

To examine if immune cell changes are present in early stages of Dementia with Lewy Bodies (DLB).

**Background:**

Evidence for inflammation in synucleinopathies has been building. An extensive number of studies have documented inflammatory changes in Parkinson’s disease (PD), but much less is known about DLB. Post-mortem DLB brains show T-cell infiltration, and flow cytometry and cytokine/chemokine measures in DLB subjects have suggested the presence of inflammatory responses in DLB. However, there is limited understanding if immune cell changes are present at the earliest stages of DLB.

**Methods:**

We recruited a small, exploratory cohort of nine subjects with Mild Cognitive Impairment—Lewy Bodies (MCI-LB) as part of the broader Alabama Udall cohort study, which is focused on the role of inflammation in early PD. The MCI-LB cohort was compared to subjects with *de novo* PD (*n* = 64) and healthy controls (*n* = 70) from the larger Alabama Udall cohort. Subjects underwent clinical assessments, including Movement Disorder Society-United Parkinson’s Disease rating scale (MDS-UPDRS), and comprehensive cognitive assessment. Blood was obtained for flow cytometry and cytokine/chemokine analyses.

**Results:**

Demographics and medical history were comparable between groups. Significant differences in University of Pennsylvania Smell Identification Test, Schwab and England Activities of Daily Living, and MDS-UPDRS scores were observed between the groups. MCI-LB subjects had poorer cognitive composite scores compared to PD participants. MCI-LB subjects demonstrated a robust 60% reduction in mature neutrophils compared to healthy controls.

**Conclusion:**

Our data show changes in innate immune cells in MCI-LB compared to healthy controls in this small exploratory study. A larger cohort study is needed to validate these findings.

## Introduction

The role of inflammation in neurodegenerative disorders is increasingly recognized. Activation of innate and adaptive arms of the immune system has been observed in Parkinson’s disease (PD). Pro-inflammatory changes have been observed at early stages of PD, suggesting that inflammation is important early in the disease process. Studies suggest that pro-inflammatory measures are linked to motor and cognitive impairment in PD (1–3). Inflammatory changes have also been observed in patients with Dementia with Lewy Bodies (DLB) (4, 5), which shares clinical and pathological features as seen in PD, including the key hallmark of Lewy Bodies and Lewy neurites. Neuroinflammation in DLB has been suggested by PET imaging with the TSPO ligand ^11^C-PK11197, which binds a mitochondrial protein found in microglia and astrocytes (6, 7). Changes in circulating immune cells, with a lower relative number of CD4^+^ T-cells and CD19^+^HLA-DR^+^ activated B-cells, are seen in DLB compared with AD (8); these flow cytometry changes suggest reduced activation or exhaustion of T-cells and B-cells. Elevations of pro-inflammatory cytokines/chemokines have also been observed in blood from DLB subjects compared to controls (5, 7–11). Plasma cytokines/chemokines are associated with disease severity in DLB in some studies but not all (5, 8–10).

These data suggest that inflammatory changes contribute to the progression of DLB, yet it is not clear whether inflammation plays a role at the earliest stages of DLB. A few studies have shown possible increases in plasma cytokines in early DLB with a reduction in these levels over time (5, 9, 12). Brain imaging suggests that TSPO binding potential is higher in mild DLB compared to advanced DLB (7). To further examine this question, we recruited a small cohort of subjects with Mild Cognitive Impairment—Lewy Bodies (MCI-LB) as part of the broader Alabama Udall cohort study, which has been examining the role of inflammation in early, *de novo* PD. We defined this early stage of DLB using criteria similar to those outlined by McKeith et al. (13). Subjects who fulfilled these criteria underwent the same clinical evaluation as control and PD subjects enrolled in the Alabama Udall cohort (14), including extensive cognitive testing that fulfill the Movement Disorders Society (MDS) task force criteria for Level II diagnosis of mild cognitive impairment (15). Blood samples from subjects were used for plasma cytokine/chemokine measures and peripheral immune cell phenotyping by flow cytometry. Here we show that MCI-LB subjects revealed immune cell changes compared to healthy controls as observed by immune cell profiling in this small, exploratory study.

## Methods

### Standard protocol approvals, registrations, and patient consents

Healthy control, *de novo* PD, and MCI-LB subjects were enrolled between November 2018 and April 2023 at the University of Alabama at Birmingham. The study was approved by the Institutional Review Board at UAB, and full written informed consent was obtained on each participant.

### Participants

Nine subjects with MCI-LB were enrolled in this exploratory arm of the Alabama Udall study. Criteria for MCI-LB subjects were based on the DSM-5 definition of Probable Mild Neurocognitive Disorder with Lewy Bodies (16) and the McKeith et al. (13) criteria. Inclusion criteria included at least two of the following five core features (fluctuating cognition; visual hallucinations, stereotyped illusions, and/or recurring delusions; REM sleep behavior disorder, Parkinsonism; and impaired visuospatial performance). Exclusion criteria for MCI-LB subjects included clinical diagnosis of moderate or greater dementia or treatment with dopaminergic therapies for Parkinsonism.

64 subjects with newly diagnosed PD and 70 age and sex-matched healthy controls (HC) were enrolled in the Alabama Udall cohort. Inclusion and exclusion criteria for HC and PD subjects have been previously described (14). Briefly, criteria for PD participants included PD diagnosis using UK Brain Bank criteria (17) of less than 2 years at time of enrollment, no previous treatment with PD medications, Hoehn and Yahr stage I-III, and minimum age of 40. Diagnosis with autoimmune or inflammatory disorders, active treatment with immunosuppressants, and serious comorbidity potentially affecting study participation were exclusion criteria for all subjects in the study.

### Clinical evaluation

After consent, participants underwent an extensive battery of clinical assessment over one or two visits. Clinical assessments for all subjects included demographics, medical history, immune disorder questionnaire, vaccination history, medication use, family history, behavioral history, PD Screening Questionnaire, vital signs, neurological examination, Epworth Sleepiness Scale, Hamilton Anxiety and Depression scales, Movement Disorder Society-United Parkinson’s Disease rating scale (MDS-UPDRS), Modified Schwab and England Activities of Daily Living, Montreal Cognitive Assessment (MoCA), Parkinson’s Disease Quality of Life Questionnaire (PDQ-39), Rapid Eye Movement Behavior Disorder Questionnaire, Pittsburgh Sleep Quality Index (PSQI), Scales for Outcomes in Parkinson’s Disease – Autonomic Dysfunction (SCOPA-AUT), and University of Pennsylvania Smell Identification Test (UPSIT). Subjects also participated in comprehensive cognitive testing meeting the Level II diagnostic mild cognitive impairment (MCI) assessment criteria recommended by the MDS task force (15). A minimum of two tests were performed in each of seven cognitive domains: attention, language, verbal memory, visual memory, executive function, visuospatial ability, and processing speed. The tests performed were Wechsler Adult Intelligence Scale - Fourth Edition (WAIS-IV) Digit Span and Letter-Number Sequencing (18); Hopkins Verbal Learning Test - Revised (HVLT-R) (19); 10/36 Spatial Recall Test (20); Judgment of Line Orientation (JLO) (21); Hooper Visual Organization Test (22); Boston Naming Test (BNT) (23); Animal Naming (24); Delis-Kaplan Executive Function System (DKEFS) Color/Word Interference (25); and Trail Making Test (TMT) (26). For each cognitive domain, normally distributed z-scores were calculated using the best available normative mean and standard deviation. Normative groups were stratified by age and education whenever possible. The cognitive composite score is the average of all domain z-scores.

### Biospecimen collection

Blood samples from each participant was used for flow cytometry studies, complete blood count, plasma collection, and DNA isolation. Unused plasma and DNA samples were banked at the UAB Center for Clinical and Translational Science (CCTS) Sample Processing and Analysis Network (SPAN) and at the NINDS BioSEND program.

### Cytokine/chemokine analysis

Plasma cytokine and chemokine levels from participants was determined with the V-PLEX Proinflammatory Panel 1 and the V-PLEX Chemokine Panel 1 using the MSD SECTOR Imager 2,400 at the UAB Diabetes Research Center Human Physiology Core, as previously described (14).

### Flow cytometry

Immune cell profiling by flow cytometry was performed as previously described (14). 500 μL of fresh peripheral blood was blocked with Human TruStain FcX (BioLegend, San Diego, CA) to prevent non-specific Fc receptor binding. Red blood cells (RBCs) were lysed using RBC Lysis Buffer (BioLegend San Diego, CA). After washing, cells were fixed with 2% paraformaldehyde, and cell viability was assessed using the Aqua Live/Dead Kit (ThermoFisher Scientific). Cells were labeled with specific fluorochrome-conjugated monoclonal antibodies to immune cell surface markers. Antibodies used were from BioLegend: anti-CD45 Pacific Blue (clone HI30); anti-CD3 Brilliant Violet 605 (clone OKT3); anti-CD4 PE (clone OKT4); anti-CD4 eFluor 450 (clone OKT4); anti-CD8α FITC (clone HIT8a); anti-CD14 FITC (clone HCD14); anti-CD16 APC (clone 3G8); anti-CD19 Brilliant Violet 650 (clone HIB19); anti-CD27 Brilliant Violet 421 (clone O323); anti-CD38 PerCP-Cyanine5.5 (clone HB-7); anti-CD45RA APC (clone HI100); anti-CD56 BUV395 (clone 14. G2a); anti-CD62L APC/Cyanine7 (clone DREG-56); anti-CD66b PE (clone G10F5); and anti-CCR7 PE-Cy7 (clone G043H7). Cells were incubated with antibodies (diluted at 1:100) for 20–30 min at room temperature. Flow cytometry was performed using a FACSymphony (BD Biosciences), and immune cell subset analysis was conducted with FlowJo software (Tree Star, Inc., Ashland, OR).

### Statistical analysis

Study sample characteristics were described using means and standard deviations for continuous variables and frequencies (percentages) for categorical variables. Differences between groups for the entire study sample were analyzed using independent samples *t* tests (two groups) or one-way ANOVA (three groups) for continuous variables and Pearson chi-square tests (or Fisher’s exact tests) for categorical variables. For cytokine and chemokine analyses, samples with levels below the detectable range were imputed as a value of equal to half the limit of detection (LoD) in the analysis. Sensitivity analyses were also performed where these values were excluded, and where these values were imputed with 0 and with a value equal to the LoD. Differences in neuropsychological tests (cognitive composite and domain z-scores) and flow cytometry measurements between MCI-LB and PD subjects were examined using linear regression analysis. For flow cytometry measurements and blood counts, due to concerns about effects of sample size imbalance on estimates, we also performed sensitivity analyses using 1:1 matching between MCI-LB and HC subjects for analysis, based on an exact match for gender and nearest neighbor match on age (Supplementary Table 6), and using nonparametric tests (Supplementary Table 7). In keeping with the exploratory nature of our study, our primary analysis did not correct for multiple comparisons, but sensitivity analyses using a Hochberg step-down procedure were also performed (27). Statistical analyses were performed using version 4.2.0 Patched of the R programming environment (28). Matched samples were identified using version 4.5.5 of the *MatchIt* package for *R* (29).

### Data sharing

Clinical data have been uploaded to the NINDS Data Management Resource. DNA, plasma, and CSF samples have been stored at the NINDS BioSEND repository. Additional data are available to investigators upon request.

## Results

### Referral data

**Figure 1:**
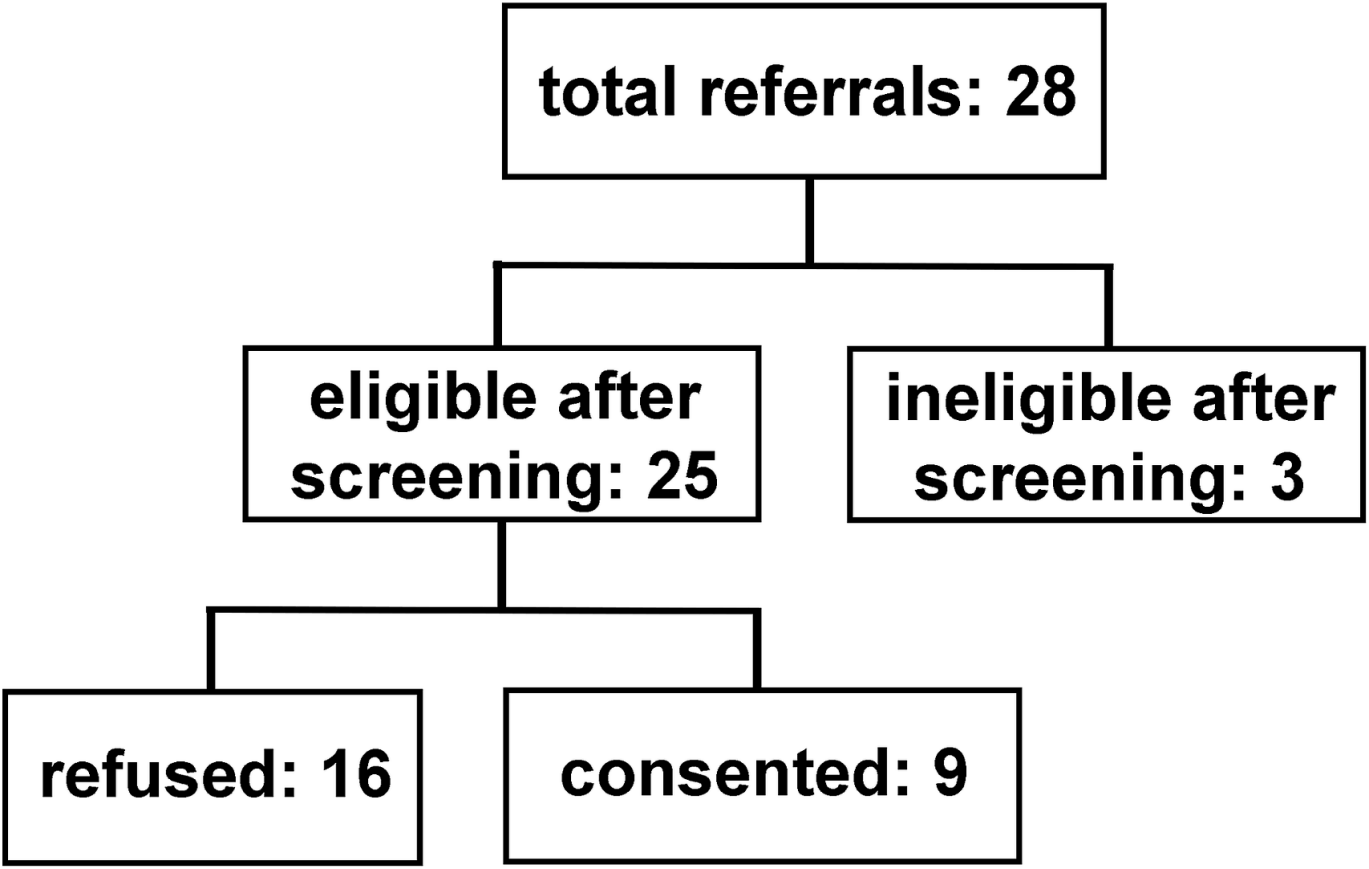
Flow chart of enrollment of MCI-LB arm of the Alabama Udall cohort.

28 potential subjects for the MCI-LB arm were referred for this study between September 2020 and November 2022 (Figure 1). 25 were eligible after screening. Out of the eligible candidates, 16 refused to participate or did not respond to calls for scheduling visits. Nine subjects ultimately consented and enrolled in the study. Enrollment of the main Alabama Udall cohort of HC and PD participants has been previously described (14).

### Demographic and clinical features

The nine MCI-LB subjects were compared to the 70 HC and 64 *de novo* PD subjects enrolled in the broader Alabama Udall cohort. Baseline demographics (Table 1), medical history (Supplementary Table 1), and family history (Supplementary Table 2) were generally comparable between groups. The MCI-LB group showed a higher percentage of disabled employment status (Table 1). This group also showed a higher rate of renal disease and COVID-19 infection (Supplementary Table 1). The higher rate of COVID-19 infection is likely related to the fact that these subjects were enrolled after the onset of the COVID-19 pandemic, while a large percentage of the HC and PD participants were enrolled prior to the spring of 2020, when the COVID-19 pandemic started in the United States. With regard to family history, the MCI-LB group also showed a higher rate of family history of PD and schizophrenia (Supplementary Table 2). Vaccination history at baseline also differed, with higher rates in the MCI-LB group (Supplementary Table 3), likely related to enrollment of these participants after the COVID-19 vaccination was available.

**Table 1:** Demographics.

	Control	PD	MCI-LB	*p*
	*N = 70*	*N = 64*	*N = 9*	
Age, years	64.1 (9.34)	66.0 (8.61)	63.1 (12.1)	0.389
Gender				0.099
Female	40 (57.1%)	26 (40.6%)	3 (33.3%)	
Male	30 (42.9%)	38 (59.4%)	6 (66.7%)	
Ethnicity				0.472
Hispanic or Latino	0 (0.00%)	1 (1.56%)	0 (0.00%)	
Not Hispanic or Latino	68 (97.1%)	63 (98.4%)	9 (100%)	
Not reported	2 (2.86%)	0 (0.00%)	0 (0.00%)	
American Black	5 (7.14%)	5 (7.81%)	0 (0.00%)	1.000
European ancestry	65 (92.9%)	59 (92.2%)	9 (100%)	1.000
Education level				
High school graduate	11 (15.7%)	3 (4.69%)	0 (0.00%)	
Some college, no degree	9 (12.9%)	13 (20.3%)	3 (33.3%)	
Associate degree: occupational, technical, or vocational program	4 (5.71%)	3 (4.69%)	0 (0.00%)	
Associate degree: academic program	5 (7.14%)	4 (6.25%)	2 (22.2%)	
Bachelors degree	19 (27.1%)	16 (25.0%)	1 (11.1%)	
Masters degree	16 (22.9%)	19 (29.7%)	0 (0.00%)	
Professional school degree	6 (8.57%)	3 (4.69%)	3 (33.3%)	
Doctoral degree	0 (0.00%)	3 (4.69%)	0 (0.00%)	
Employment status				**<0.001**
Working now	32 (45.7%)	22 (34.4%)	4 (44.4%)	
Retired	34 (48.6%)	36 (56.2%)	1 (11.1%)	
Disabled, permanently or temporarily	0 (0.00%)	1 (1.56%)	4 (44.4%)	
Keeping house	3 (4.29%)	2 (3.12%)	0 (0.00%)	
Other	1 (1.43%)	3 (4.69%)	0 (0.00%)	

The bold values refer to *p* ≤ 0.05.

As expected, there were significant differences between HC, PD, and MCI-LB participants with regard to clinical assessment scales, including the UPSIT, Schwab and England Activities of Daily Living, Hamilton Anxiety and Depression, SCOPA-AUT, and MDS-UPDRS scores (Table 2). Both PD and MCI-LB groups showed lower UPSIT scores and Schwab and England ADL scores and higher SCOPA-AUT and UPDRS scores compared to HC.

**Table 2:** Clinical assessment scores at baseline.

	Control	PD	MCI-LB	*p*	N
	*N = 70*	*N = 64*	*N = 9*		
PDQ-39 total	n/a	16.9 (15.9)	38.7 (25.1)	**0.001**	73
MoCA total points	27.4 (1.75)	25.5 (3.68)	21.1 (7.54)	**<0.001**	138
UPSIT total score	32.5 (4.78)	22.8 (7.86)	25.6 (10.5)	**<0.001**	142
Schwab & England ADL score	97.4 (5.57)	91.2 (9.92)	82.2 (8.33)	**<0.001**	143
Epworth sleepiness scale total	4.99 (3.55)	5.75 (4.57)	8.56 (6.41)	0.055	143
HAM-A total score	2.74 (4.81)	3.30 (2.95)	11.6 (5.29)	**<0.001**	143
HAM-D total score	3.10 (2.28)	2.45 (2.58)	7.44 (4.16)	**<0.001**	143
SCOPA-AUT score	5.46 (4.26)	10.1 (6.41)	12.9 (5.35)	**<0.001**	143
PSQI total	5.16 (3.39)	4.91 (3.62)	8.22 (4.35)	**0.034**	143
UPDRS total	8.59 (5.89)	43.1 (14.2)	52.2 (29.2)	**<0.001**	142
UPDRS Part 1	3.51 (3.26)	6.37 (5.30)	16.3 (7.28)	**<0.001**	142
UPDRS Part 2	0.66 (1.18)	5.46 (3.94)	9.44 (8.53)	**<0.001**	142
UPDRS Part 3	4.41 (3.55)	31.2 (9.16)	26.4 (18.7)	**<0.001**	142
UPDRS Part 4	0.00 (0.00)	0.00 (0.00)	0.00 (0.00)	.	142

The bold values refer to *p* ≤ 0.05.

### MCI-LB subjects show greater cognitive impairment compared to early PD subjects

We next evaluated the cognitive profile of HC, PD, and MCI-LB participants. MCI-LB subjects had the lowest MoCA scores (Table 2). Differences in cognitive composite scores and in individual cognitive subdomains were observed among the three groups (Table 3). Linear regression analysis revealed that MCI-LB participants did more poorly in attention, verbal memory, general memory, language, processing speed, and executive subdomains compared to PD participants (Table 3). These differences suggest that the criteria used to recruit MCI-LB subjects were able to distinguish these MCI-LB subjects from *de novo* PD subjects, who have less cognitive impairment compared to subjects with early DLB.

**Table 3:** Neuropsychological testing at baseline.

	Control	PD	MCI-LB	*p*	N
	*N = 68*	*N = 62*	*N = 9*		
Attention Z Score	0.47 (0.76)	0.32 (0.86)	−0.50 (0.74)	**0.011**	136
Verbal Memory Z Score	0.22 (0.93)	−0.01 (1.03)	−1.31 (0.88)	**<0.001**	138
Visual Memory Z Score	0.27 (0.82)	−0.01 (0.90)	−0.47 (1.05)	**0.034**	137
General Memory Z Score	0.20 (0.68)	0.04 (0.73)	−0.76 (0.86)	**0.001**	139
Visual Spatial Z Score	0.33 (0.71)	0.04 (0.94)	−0.24 (0.75)	**0.047**	138
Language Z Score	0.50 (0.69)	0.39 (0.74)	−0.36 (0.68)	**0.004**	138
Processing Speed Z Score	0.26 (0.56)	0.05 (0.87)	−0.97 (1.18)	**<0.001**	139
Executive Function Z Score	0.33 (0.72)	0.05 (0.94)	−1.06 (0.96)	**<0.001**	136
Cognitive Composite 7	0.34 (0.47)	0.17 (0.58)	−0.33 (0.45)	**0.009**	132
Cognitive Composite 5	0.37 (0.48)	0.21 (0.60)	−0.21 (0.29)	**0.031**	132

	Estimate (MCI-LB vs. PD)	95% CI	*p*	N	R^2^
Attention Z Score	−0.82	−1.48–−0.16	**0.015**	68	0.082
Verbal Memory Z Score	−1.30	−2.05–−0.56	**0.001**	70	0.148
Visual Memory Z Score	−0.46	−1.13–0.21	0.180	70	0.026
General Memory Z Score	−0.79	−1.31–−0.27	**0.003**	71	0.115
Visual Spatial Z Score	−0.28	−0.96–0.40	0.415	70	0.010
Language Z Score	−0.75	−1.26–−0.23	**0.005**	71	0.104
Processing Speed Z Score	−1.03	−1.67–−0.39	**0.002**	71	0.125
Executive Function Z Score	−1.11	−1.84–−0.38	**0.003**	68	0.118
Cognitive Composite 7	−0.50	−1.02–0.02	0.061	65	0.053
Cognitive Composite 5	−0.42	−0.96–0.11	0.123	65	0.036

The bold values refer to *p* ≤ 0.05.

### Immune cell changes are observed in MCI-LB

We examined the percentages of innate immune cells monocytes (CD56^−^CD66b^−^CD3^−^CD19^−^ cells), NK cells (CD3^−^CD19^−^ cells), and neutrophils (CD66b^+^ cells) and adaptive immune cells (CD4^+^ T-cells, CD8^+^ T-cells, and CD19^+^ B-cells) in the MCI-LB subjects and HCs to determine whether immune changes are observed in early stages of DLB. We initially compared all 70 HC to the MCI-LB cohort. Additionally, given the mismatch in the number of participants in the two groups, we repeated our analysis in which we compared the MCI-LB cohort to a similar number of age- and sex-matched HC participants. With regard to complete blood counts and differential, MCI-LB subjects showed higher total neutrophil count compared to all HC in unadjusted analyses, although this difference was not statistically significant after controlling for multiple comparisons or in the matched HC comparison (Supplementary Table 4). Of note, one MCI-LB blood sample was not able to be processed for CBC. Plasma cytokines and chemokines did not differ between MCI-LB and HC groups (Supplementary Table 5).

**Figure 2:**
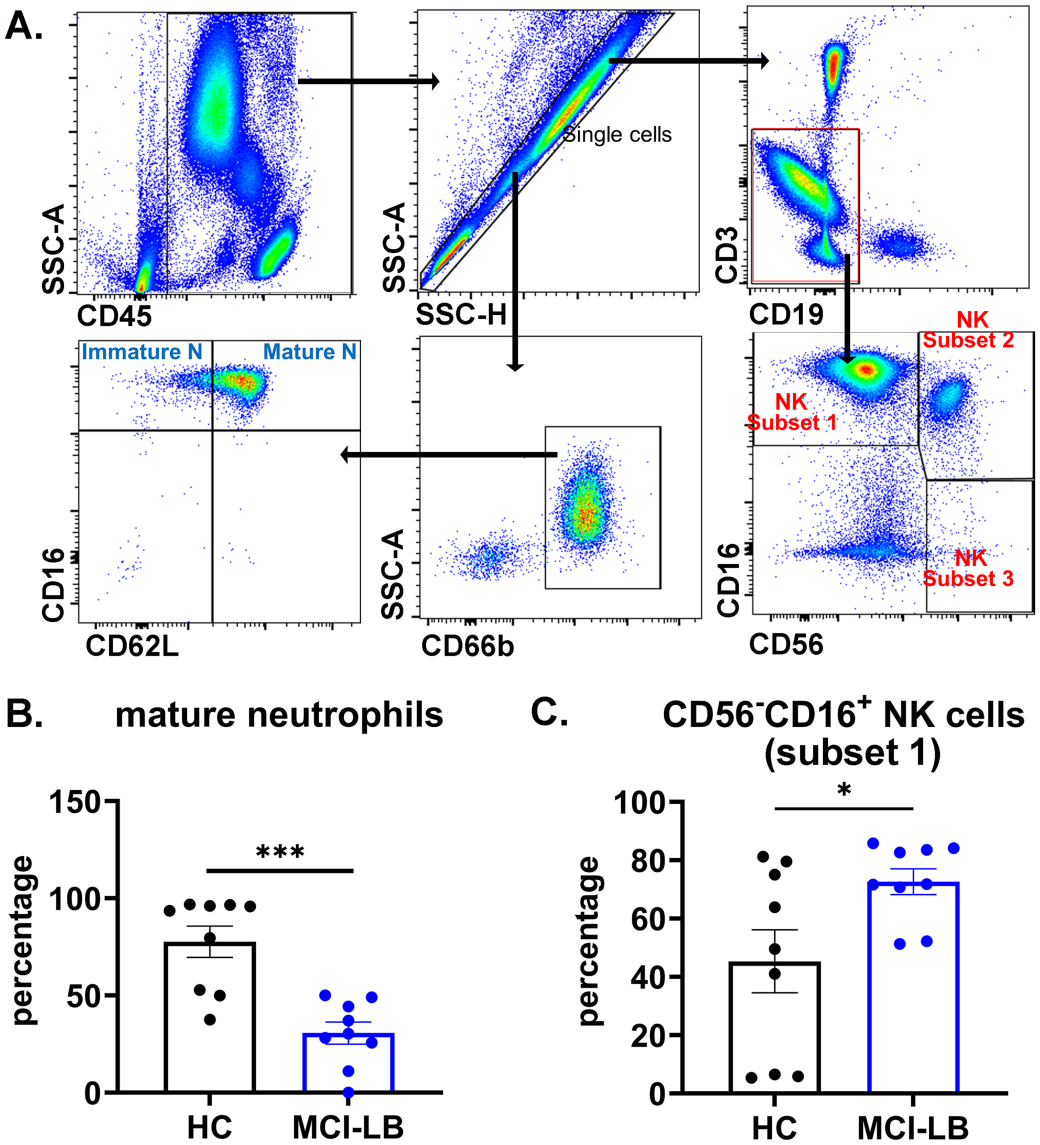
Alterations in mature neutrophils and natural killer (NK) cells in MCI-LB. **(A)** Gating strategy of neutrophil subsets and NK subsets. Blood cells were stained with conjugated antibodies for immune cell surface markers. CD45^+^ singlet cells were analyzed for immune cell subsets by flow cytometry. Parental neutrophils were gated as CD66b^+^. Immature neutrophils were gated as CD16^+^CD62L^−^, and mature neutrophils were gated as CD16^+^CD62L^+^. Parental NK cells were gated as CD3^−^CD19^−^. NK cell subset 1 was gated as CD56^−^CD16^+^, NK cell subset 2 was gated as CD56^dim^CD16^+^, and NK cell subset 3 was gated as CD56^bright^CD16^−^. **(B)** Mature neutrophils (CD16^+^CD62L^+^) in matched HC and MCI-LB participants. ***p < 0.001. **(C)** CD56^−^CD16^+^ NK cell subset 1 in matched HC and MCI-LB participants. * *p* < 0.05.

We did observe several differences in immune cell populations as determined by flow cytometry. The most dramatic change in MCI-LB participants was a ~ 60% reduction in mature neutrophils when compared to all HC subjects (Table 4) or matched HC subjects (Supplementary Table 6; Figure 2). MCI-LB participants also showed a significant increase in CD56^−^CD16^+^ NK cell subset 1 when compared to all HC with correction for multiple comparisons (Table 4). This difference was also observed when MCI-LB participants were compared to matched HC participants, but was no longer statistically significant after adjusting for multiple comparisons in the matched analysis (Supplementary Table 6; Figure 2).

**Table 4:** Flow cytometry measurements at baseline in control and MCI-LB subjects.

	HC	MCI-LB	Estimate (MCI-LB vs. HC)	95% LCI	95% UCI	*p*	*p-*adjusted	N
Classical monocytes	61.2 (28.0)	53.1 (27.5)	−8.05	−27.82	11.71	0.429	0.978	76
Intermediate monocytes	8.09 (5.86)	8.63 (9.15)	0.54	−3.92	4.99	0.868	0.978	76
Non-classical monocytes	10.8 (14.3)	13.7 (9.76)	2.81	−7.00	12.62	0.460	0.978	76
Tregulatory cells	23.9 (20.8)	21.4 (20.9)	−2.54	−17.19	12.12	0.739	0.978	78
Non-Tregulatory cells	67.7 (21.6)	73.8 (22.0)	6.16	−9.14	21.46	0.447	0.978	78
Th2 cells	14.9 (14.1)	14.7 (19.6)	−0.20	−10.64	10.25	0.977	0.978	78
CXCR3^+^ memory B-cells	9.31 (8.02)	17.7 (15.6)	8.42	1.91	14.94	0.149	0.978	74
Bregulatory (Breg) cells	5.46 (4.14)	13.1 (10.7)	7.63	3.87	11.38	0.066	0.978	74
Th1 cells	20.1 (10.9)	23.2 (12.1)	3.10	−4.70	10.90	0.483	0.978	78
Th17 cells	6.98 (4.52)	7.69 (12.1)	0.71	−3.38	4.80	0.865	0.978	78
Th1/Th17 cells	7.44 (5.16)	7.96 (7.99)	0.52	−3.38	4.42	0.853	0.978	78
CD4^+^ central memory T-cells	23.1 (16.0)	24.8 (16.3)	1.71	−9.58	13.01	0.772	0.978	78
CD4^+^ naive	36.2 (18.9)	34.7 (15.3)	−1.57	−14.66	11.51	0.783	0.978	78
CD4^+^ effector memory T-cells	30.5 (19.1)	35.3 (9.14)	4.81	−8.14	17.76	0.223	0.978	78
CD8 + central memory T-cells	8.77 (10.6)	8.24 (8.81)	−0.54	−7.89	6.81	0.869	0.978	78
CD8^+^ naive	26.0 (21.7)	15.6 (9.84)	−10.47	−25.10	4.17	**0.021**	0.507	78
CD8^+^ terminally differentiated T-cells	32.4 (21.3)	34.3 (16.2)	1.89	−12.79	16.57	0.758	0.978	78
CD8^+^ effector memory T-cells	32.8 (23.1)	41.9 (15.2)	9.14	−6.69	24.98	0.138	0.978	78
Switched memory B-cells	16.1 (15.3)	11.1 (7.22)	−5.01	−15.35	5.32	0.117	0.978	74
Non-switched memory B-cells	8.13 (10.8)	6.82 (7.11)	−1.31	−8.70	6.08	0.637	0.978	74
Naïve B-cells	61.9 (23.8)	68.0 (18.2)	6.16	−10.34	22.66	0.379	0.978	74
Double negative B-cells	13.8 (16.8)	14.0 (15.1)	0.16	−11.60	11.91	0.978	0.978	74
NK subset 1 (CD56^−^CD16^+^)	50.3 (31.0)	72.6 (13.2)	22.36	1.42	43.29	**0.001**	**0.022**	76
NK subset 2 (CD56^dim^CD16^+^)	10.1 (13.8)	10.2 (6.97)	0.12	−9.23	9.46	0.968	0.978	76
NK subset 3 (CD56^bright^CD16^−^)	1.50 (1.97)	0.81 (0.43)	−0.70	−2.02	0.63	**0.016**	0.402	76
Immature neutrophils	22.4 (24.1)	16.9 (14.8)	−5.49	−21.94	10.97	0.354	0.978	77
Mature neutrophils	75.3 (24.1)	30.6 (16.9)	−44.66	−61.24	−28.07	**<0.001**	**<0.001**	77

Each cell subset is shown as a percentage of the total parental cells, i.e., percentage of innate immune cells monocytes (CD56^−^CD66b^−^CD3^−^CD19^−^ cells), NK cells (CD3^−^CD19^−^ cells), neutrophils (CD66b^+^ cells), or adaptive immune cells (CD4^+^ T-cells, CD8^+^ T-cells, and CD19^+^ B-cells), respectively. For example, each monocyte subset (classical, intermediate, or non-classical) are shown as the percentage of total monocyte populations (gated as CD56^−^CD66b^−^CD3^−^CD19^−^ cells). LCI, lower confidence interval. UCI, upper confidence interval. P adjusted using Hochberg correction. The bold values refer to *p* ≤ 0.05.

Sensitivity analysis examined the immune cell profiles controlling for covariates including COVID infection, COVID vaccination, and renal disease as factors that were significantly different between the HC and PD groups. We found that mature neutrophils remained significantly different between the two groups (Supplementary Table 8). Interestingly, in this analysis, we also observed an increase in regulatory B-cells (Bregs) in the MCI-LB group compared to HC when including these covariates in the analysis (Supplementary Table 8).

## Discussion

In this study, we enrolled subjects at an early stage of DLB and compared their cognitive profiles and immune cell profiles to HC and PD participants. We found that using these criteria, we were able to recruit a cohort of MCI-LB subjects that showed a more impaired cognitive profile compared to early PD subjects. Not surprisingly, the MCI-LB subjects showed a worse cognitive composite score compared to PD subjects. The MCI-LB group also showed poorer performance in attention, verbal memory, general memory, language, processing speed, and executive subdomains. While it is well established that patients can demonstrate cognitive deficits at early stages of PD (14, 30–32), the differences in cognitive testing observed in the MCI-LB group support the view that the MCI-LB subjects represent a different subset of Lewy Body disease compared to the *de novo* PD subjects in our Udall study.

The MCI-LB group consistently demonstrated a significant decrease in mature neutrophils. Neutrophils, traditionally the first-line defenders against infections, play critical roles in neurodegenerative diseases such as Alzheimer’s Disease (AD) and PD (33–35). In AD models, neutrophils infiltrate the central nervous system and accumulate around amyloid-beta plaques (36, 37). Neutrophil hyperactivation is associated with disease progression in AD subjects (38). Elevated neutrophils have been observed in PD, and some studies suggest that increased neutrophils may be associated with disease severity (39–42). Neutrophils contribute to pathology by releasing reactive oxygen species, proteolytic enzymes like matrix metalloproteinases, and pro-inflammatory cytokines (e.g., IL-1β, TNF-*α*), as well as forming neutrophil extracellular traps, which disrupt the blood–brain barrier (33–35). In the MCI-LB patients, the decrease in mature neutrophils compared to HC is distinct from observations in AD and PD. This may reflect a different function of mature neutrophils in MCI-LB. Whether this pattern would continue with later stages of DLB will need to be examined in future studies.

We also observed potential, less robust changes for other immune cells in our exploratory cohort, including NK cells, yet these data require replication in future studies. NK cell subsets have traditionally been defined by the level of expression of two cell surface proteins, CD56 and CD16 (43). The CD56^−^CD16^+^ NK cell subset 1 has been referred to as exhausted or dysfunctional, with reduced cytokine production and cytotoxic function, and is associated with impaired immune responses (44–47). We observed a possible increase in CD56^−^CD16^+^ NK cell subset 1 percentages in our MCI-LB group when comparing to all HC participants or matched HC participants. This increase suggests that NK cells with impaired cytokine production and cytotoxicity may be prominent in the early stages of DLB. Of note, when we included COVID infection, renal disease, and COVID vaccination in our covariate analysis, the difference in NK cell subset 1 between MCI-LB and HC groups was no longer statistically significant, likely related to the small sample size for the MCI-LB group. Replication with a larger MCI-LB sample size in the future will help determine the generalizability of these NK cell findings in early DLB.

In terms of other peripheral immune cells, alterations in T-cell and B-cell subpopulations have been observed in DLB, yet these studies were focused on more advanced disease. Using flow cytometry, Amin *et al.* observed a decrease in CD4^+^ T-cell and B-cell subsets (8). An increase in T-cells has been observed in postmortem DLB brain tissue (48–51). We observed a potential increase in CD19^+^CD24^hi^CD38^hi^ Bregs in the MCI-LB group compared to the full HC group, but this was observed only after including covariates. Bregs exert immunosuppressive functions by producing anti-inflammatory cytokines, such as IL-10, TGF-*β* and IL-35 (52, 53). Further research using larger sample sizes and longitudinal study design is needed to understand specific changes in T and B-cell populations in early DLB.

The biggest limitation of this study was the low number of enrollees in the MCI-LB group which limited the statistical power to determine changes in immune cell parameters between our control and MCI-LB groups. The most consistent change regardless of our analysis approach was in mature neutrophils. We also observed potential changes in NK cell populations and Bregs, among others, but changes in these immune cell populations should be viewed more cautiously as they did not necessarily remain significant when adjusting for multiple comparisons or covariates. Validation of our findings with a larger cohort will be important to fully determine which immune cell changes are observed at the earliest stages of DLB, but identification of MCI-LB subjects remains a significant challenge for replication studies. Despite an active search for these subjects in the movement disorders, memory disorders, and sleep medicine clinics at UAB, we found it difficult to identify subjects who fulfilled the criteria and who were willing to participate. Over the enrollment period, we did slightly modify our initial criteria to eliminate the requirements that cognitive symptoms precede any Parkinsonism and that the duration of cognitive symptoms was less than 2 years. This alteration in the initial criteria could have added to variability in our MCI-LB cohort, perhaps allowing for the inclusion of some patients with PD-MCI. Better biomarkers are still needed in order to identify the prodromal stages of DLB.

In conclusion, this initial small, exploratory study provides preliminary evidence for immune cell alterations in MCI-LB subjects. Replication of these findings in a larger cohort are indicated to confirm these findings and to determine the relevance and applicability of these findings to early DLB. Longitudinal follow-up of this cohort and larger cohorts would be important in order to understand whether such immune changes at baseline may be predictive of the rate of cognitive decline. Validation of these immune cell changes from our small study in larger cohorts could point to a therapeutic target in MCI-LB.

## Data Availability

The original contributions presented in the study are included in the article/Supplementary material, further inquiries can be directed to the corresponding author.
