# Supplementary material for: Inflammatory changes in early dementia with Lewy bodies
Source: Front Neurol. 2025 Aug 26;16:1640614. doi: 10.3389/fneur.2025.1640614 (PMC12417133; doi:10.3389/fneur.2025.1640614)
Supplement: Supplementary file 1 [file Table_1.docx]

|  | **Control** | **PD** | **MCI-LB** | ***p*** |
| --- | --- | --- | --- | --- |
|  | ***N=70*** | ***N=64*** | ***N=9*** |  |
| **Hypertension** | 33 (47.1%) | 33 (51.6%) | 4 (44.4%) | 0.832 |
| **Diabetes mellitus (childhood onset)** | 0 (0%) | 0 (0%) | 0 (0%) | . |
| **Diabetes mellitus (adult onset)** | 8 (11.4%) | 8 (12.5%) | 2 (22.2%) | 0.614 |
| **Myocardial infarction** | 3 (4.29%) | 1 (1.56%) | 0 (0.00%) | 0.708 |
| **Congestive heart failure** | 0 (0%) | 0 (0%) | 0 (0%) | . |
| **Arrhythmia/atrial fibrillation** | 5 (7.14%) | 4 (6.25%) | 1 (11.1%) | 0.754 |
| **Hypercholesterolemia** | 33 (47.1%) | 34 (53.1%) | 5 (55.6%) | 0.771 |
| **Lung disease** | 0 (0.00%) | 1 (1.56%) | 1 (11.1%) | 0.060 |
| **Thyroid disease** | 11 (15.7%) | 7 (10.9%) | 0 (0.00%) | 0.432 |
| **Liver disease** | 2 (2.86%) | 1 (1.56%) | 0 (0.00%) | 1.000 |
| **Renal disease** | 1 (1.43%) | 3 (4.69%) | 2 (22.2%) | **0.032** |
| **Peripheral vascular disease** | 1 (1.43%) | 1 (1.56%) | 0 (0.00%) | 1.000 |
| **Stroke or TIA** | 2 (2.86%) | 6 (9.38%) | 0 (0.00%) | 0.256 |
| **Seizure** | 0 (0.00%) | 1 (1.56%) | 0 (0.00%) | 0.510 |
| **Gaucher disease** | 0 (0%) | 0 (0%) | 0 (0%) | . |
| **Multiple sclerosis** | 0 (0%) | 0 (100%) | 0 (0%) | . |
| **Cancer** | 13 (18.6%) | 23 (35.9%) | 3 (33.3%) | 0.058 |
| **COVID-19** | 1 (1.43%) | 1 (1.56%) | 2 (22.2%) | **0.020** |
| **Other** | 25 (35.7%) | 26 (40.6%) | 7 (77.8%) | 0.058 |

**Supplemental Table 1. Medical history at baseline**

**Supplemental Table 2. Family history at baseline**

|  | **Control** | **PD** | **MCI-LB** | ***p*** |
| --- | --- | --- | --- | --- |
|  | ***N=70*** | ***N=64*** | ***N=9*** |  |
| **Alzheimer's disease** | 13 (18.6%) | 18 (28.1%) | 3 (33.3%) | 0.307 |
| **Amyotrophic lateral sclerosis** | 1 (1.43%) | 3 (4.69%) | 0 (0.00%) | 0.499 |
| **Ataxia** | 0 (0%) | 0 (0%) | 0 (0%) | . |
| **Autism** | 7 (10.0%) | 7 (10.9%) | 0 (0.00%) | 0.909 |
| **Bipolar disorder** | 5 (7.14%) | 3 (4.69%) | 1 (11.1%) | 0.524 |
| **Brain aneurysm** | 8 (11.4%) | 4 (6.25%) | 2 (22.2%) | 0.174 |
| **Cancer** | 51 (72.9%) | 51 (79.7%) | 8 (88.9%) | 0.504 |
| **Dementia** | 20 (28.6%) | 25 (39.1%) | 4 (44.4%) | 0.335 |
| **Depression** | 19 (27.1%) | 23 (35.9%) | 4 (44.4%) | 0.393 |
| **Diabetes mellitus** | 28 (40.0%) | 28 (43.8%) | 4 (44.4%) | 0.927 |
| **Dystonia** | 0 (0%) | 0 (0%) | 0 (0%) | . |
| **Epilepsy** | 2 (2.86%) | 1 (1.56%) | 0 (0.00%) | 1.000 |
| **Heart Disease** | 44 (62.9%) | 45 (70.3%) | 5 (55.6%) | 0.495 |
| **Hypertension** | 41 (58.6%) | 44 (68.8%) | 6 (66.7%) | 0.506 |
| **Memory loss** | 21 (30.0%) | 25 (39.1%) | 4 (44.4%) | 0.461 |
| **Migraines** | 17 (24.3%) | 10 (15.6%) | 4 (44.4%) | 0.101 |
| **Multiple sclerosis** | 3 (4.29%) | 1 (1.56%) | 0 (0.00%) | 0.708 |
| **Muscle disease** | 0 (0.00%) | 1 (1.56%) | 0 (0.00%) | 0.510 |
| **Parkinson's disease** | 1 (1.43%) | 11 (17.2%) | 4 (44.4%) | **<0.001** |
| **Schizophrenia** | 2 (2.86%) | 0 (0.00%) | 3 (33.3%) | **0.001** |
| **Stroke** | 29 (41.4%) | 26 (40.6%) | 6 (66.7%) | 0.346 |
| **Suicide or suicide attempt** | 9 (12.9%) | 5 (7.81%) | 1 (11.1%) | 0.551 |
| **Tourette syndrome** | 1 (1.43%) | 0 (0.00%) | 0 (0.00%) | 1.000 |

**Supplementary Table 3. Vaccination history at baseline.**

|  | **Control** | **PD** | **MCI-LB** | ***p*** | |  |
| --- | --- | --- | --- | --- | --- | --- |
|  | ***N=70*** | ***N=64*** | ***N=9*** |  | |  |
| **Influenza** | 42 (60.0%) | 45 (70.3%) | 6 (66.7%) | | 0.516 | |
| **Tetanus, diphtheria, pertussis** | 0 (0.00%) | 5 (7.81%) | 0 (0.00%) | | 0.061 | |
| **Varicella** | 0 (0.00%) | 0 (0.00%) | 1 (11.1%) | | 0.063 | |
| **Zoster** | 7 (10.0%) | 8 (12.5%) | 1 (11.1%) | | 0.914 | |
| **Measles, mumps, rubella** | 70 (100%) | 64 (100%) | 9 (100%) | | . | |
| **Pneumococcal (PCV13 or PPSV23)** | 4 (5.71%) | 8 (12.5%) | 0 (0.00%) | | 0.329 | |
| **Meningococcal** | 70 (100%) | 64 (100%) | 9 (100%) | | . | |
| **Hepatitis A** | 1 (1.43%) | 1 (1.56%) | 0 (0.00%) | | 1.000 | |
| **Hepatitis B** | 70 (100%) | 64 (100%) | 9 (100%) | | . | |
| **Haemophilus influenza type B** | 70 (100%) | 64 (100%) | 9 (100%) | | . | |
| **COVID-19** | 30 (42.9%) | 14 (21.9%) | 7 (77.8%) | | **0.001** | |

**Supplemental Table 4. Complete blood counts at baseline in controls and MCI-LB subjects.**

|  | **Control**  ***N=70*** | **MCI-LB**  ***N=8*** | **Estimate**  **(MCI-LB vs HC)** | **95% LCI** | **95% UCI** | ***p*** | ***p-adjusted*** | **N** |
| --- | --- | --- | --- | --- | --- | --- | --- | --- |
| **White blood cells** | 6.00 (1.37) | 5.62 (1.31) | -0.38 | -1.40 | 0.63 | 0.456 | 0.947 | 78 |
| **Neutrophil %** | 59.1 (7.30) | 64.4 (5.42) | 5.28 | -0.03 | 10.58 | **0.031** | 0.309 | 78 |
| **Monocyte %** | 8.10 (1.95) | 8.00 (2.07) | -0.10 | -1.56 | 1.36 | 0.899 | 0.947 | 78 |
| **Lymphocyte %** | 29.3 (6.96) | 24.4 (6.82) | -4.90 | -10.06 | 0.26 | 0.088 | 0.793 | 78 |
| **Basophil %** | 0.96 (0.40) | 0.75 (0.46) | -0.21 | -0.51 | 0.09 | 0.258 | 0.947 | 78 |
| **Eosinophil %** | 2.71 (1.98) | 2.25 (1.16) | -0.46 | -1.89 | 0.96 | 0.347 | 0.947 | 78 |
| **Neutrophil to lymphocyte ratio (NLR)** | 2.21 (0.89) | 2.89 (1.05) | 0.68 | 0.00 | 1.35 | 0.118 | 0.942 | 78 |
| **Hemoglobin** | 14.1 (1.20) | 13.9 (1.44) | -0.16 | -1.07 | 0.75 | 0.765 | 0.947 | 78 |
| **Hematocrit** | 41.3 (3.28) | 41.4 (3.54) | 0.09 | -2.37 | 2.55 | 0.947 | 0.947 | 78 |
| **Platelets** | 250 (62.3) | 211 (98.7) | -39.49 | -88.93 | 9.95 | 0.302 | 0.947 | 78 |
|  | **Control**  ***N=8*** | **MCI-LB**  ***N=8*** | **Estimate**  **(MCI-LB vs HC)** | **95% LCI** | **95% UCI** | ***p*** | ***p-adjusted*** | **N** |
| **White blood cells** | 5.50 (1.76) | 5.62 (1.31) | 0.12 | -1.54 | 1.78 | 0.882 | 1.000 | 16 |
| **Neutrophil %** | 58.2 (7.42) | 64.4 (5.42) | 6.12 | -0.84 | 13.09 | 0.082 | 0.659 | 16 |
| **Monocyte %** | 7.38 (1.92) | 8.00 (2.07) | 0.62 | -1.52 | 2.77 | 0.542 | 1.000 | 16 |
| **Lymphocyte %** | 31.2 (7.09) | 24.4 (6.82) | -6.88 | -14.33 | 0.58 | 0.068 | 0.615 | 16 |
| **Basophil %** | 1.00 (0.53) | 0.75 (0.46) | -0.25 | -0.79 | 0.29 | 0.335 | 1.000 | 16 |
| **Eosinophil %** | 2.25 (1.04) | 2.25 (1.16) | 0.00 | -1.18 | 1.18 | 1.000 | 1.000 | 16 |
| **Neutrophil to lymphocyte ratio (NLR)** | 2.00 (0.70) | 2.89 (1.05) | 0.89 | -0.06 | 1.85 | 0.068 | 0.615 | 16 |
| **Hemoglobin** | 13.8 (0.94) | 13.9 (1.44) | 0.14 | -1.16 | 1.44 | 0.824 | 1.000 | 16 |
| **Hematocrit** | 40.5 (1.85) | 41.4 (3.54) | 0.87 | -2.16 | 3.91 | 0.549 | 1.000 | 16 |
| **Platelets** | 210 (70.9) | 211 (98.7) | 1.02 | -91.17 | 93.22 | 0.981 | 1.000 | 16 |

LCI: lower confidence interval. UCI: upper confidence interval. P adjusted using Hochberg correction.

**Supplemental Table 5. Plasma cytokine and chemokine levels at baseline.**

|  | **HC** | **MCI-LB** | **Estimate**  ***(MCI-LB vs HC)*** | **95% LCI** | **95% UCI** | ***p*** | ***p-adjusted*** | **N** |
| --- | --- | --- | --- | --- | --- | --- | --- | --- |
| **IFN-γ** | 5.34 (3.51) | 6.67 (1.85) | 1.33 | -1.06 | 3.71 | 0.094 | 0.976 | 77 |
| **IL-10** | 0.29 (0.14) | 0.94 (1.76) | 0.65 | 0.23 | 1.07 | 0.301 | 0.976 | 77 |
| **IL-6** | 1.10 (0.85) | 1.42 (0.51) | 0.32 | -0.26 | 0.90 | 0.125 | 0.976 | 77 |
| **IL-8** | 8.00 (4.47) | 8.13 (2.79) | 0.13 | -2.93 | 3.18 | 0.908 | 0.976 | 77 |
| **TNF-α** | 1.64 (0.47) | 1.73 (0.67) | 0.09 | -0.25 | 0.44 | 0.693 | 0.976 | 77 |
| **Eotaxin** | 235 (91.2) | 251 (62.2) | 15.41 | -50.84 | 81.66 | 0.544 | 0.976 | 75 |
| **Eotaxin-3** | 27.8 (31.5) | 28.0 (22.4) | 0.27 | -22.64 | 23.18 | 0.976 | 0.976 | 75 |
| **IP-10** | 395 (202) | 432 (161) | 37.07 | -110.67 | 184.81 | 0.564 | 0.976 | 75 |
| **MCP-1** | 99.2 (39.4) | 94.4 (31.3) | -4.80 | -33.68 | 24.07 | 0.699 | 0.976 | 75 |
| **MCP-4** | 177 (101) | 178 (54.8) | 1.19 | -71.17 | 73.56 | 0.959 | 0.976 | 75 |
| **MDC** | 1223 (463) | 1350 (259) | 127.03 | -206.76 | 460.81 | 0.259 | 0.976 | 75 |
| **MIP-1α** | 11.3 (3.56) | 12.5 (3.52) | 1.17 | -1.49 | 3.82 | 0.400 | 0.976 | 75 |
| **MIP-1β** | 73.6 (32.4) | 68.0 (19.4) | -5.61 | -28.98 | 17.75 | 0.491 | 0.976 | 75 |
| **TARC** | 377 (369) | 686 (385) | 309.76 | 33.56 | 585.96 | 0.061 | 0.848 | 75 |

Values for Control and PD are mean values (SD). Values below detection limits are imputed as LoD/2. Those cytokines and chemokines were excluded in which levels were below detection for >30% of subjects. For plasma samples, IL-2, IL-4, IL-13, IL-1β, and IL-12p70 were excluded. P adjusted using Hochberg correction.

**Supplemental Table 6. Flow cytometry for MCI-LB and age- and sex-matched controls.**

|  | **HC**  ***N=9*** | **MCI-LB**  ***N=9*** | **Estimate**  ***(MCI-LB vs HC)*** | **95% LCI** | **95% UCI** | ***p*** | ***p-adjusted*** |
| --- | --- | --- | --- | --- | --- | --- | --- |
| **Classical monocytes** | 63.9 (29.2) | 53.1 (27.5) | -10.74 | -39.10 | 17.62 | 0.434 | 0.972 |
| **Intermediate monocytes** | 9.21 (6.40) | 8.63 (9.15) | -0.59 | -8.47 | 7.30 | 0.877 | 0.972 |
| **Non-classical monocytes** | 14.7 (26.8) | 13.7 (9.76) | -1.02 | -21.18 | 19.15 | 0.917 | 0.972 |
| **T regulatory cells** | 22.5 (16.9) | 21.4 (20.9) | -1.08 | -20.07 | 17.92 | 0.906 | 0.972 |
| **Non-T regulatory cells** | 69.9 (16.8) | 73.8 (22.0) | 3.92 | -15.63 | 23.48 | 0.677 | 0.972 |
| **Th2 cells** | 11.3 (6.67) | 14.7 (19.6) | 3.37 | -11.29 | 18.03 | 0.637 | 0.972 |
| **CXCR3^+^ memory B-cells** | 15.2 (11.1) | 17.7 (15.6) | 2.56 | -11.00 | 16.11 | 0.695 | 0.972 |
| **B regulatory (Breg) cells** | 5.62 (4.18) | 13.1 (10.7) | 7.47 | -0.66 | 15.60 | 0.079 | 0.972 |
| **Th1 cells** | 20.5 (10.8) | 23.2 (12.1) | 2.70 | -8.77 | 14.16 | 0.625 | 0.972 |
| **Th17 cells** | 7.34 (2.67) | 7.69 (12.1) | 0.35 | -8.37 | 9.08 | 0.934 | 0.972 |
| **Th1/Th17 cells** | 8.83 (3.63) | 7.96 (7.99) | -0.86 | -7.07 | 5.34 | 0.773 | 0.972 |
| **CD4^+^ central memory T-cells** | 32.0 (15.5) | 24.8 (16.3) | -7.22 | -23.10 | 8.66 | 0.350 | 0.972 |
| **CD4^+^ naive** | 28.5 (9.56) | 34.7 (15.3) | 6.12 | -6.61 | 18.86 | 0.326 | 0.972 |
| **CD4^+^ effector memory T-cells** | 34.3 (15.6) | 35.3 (9.14) | 1.01 | -11.74 | 13.76 | 0.869 | 0.972 |
| **CD8+ central memory T-cells** | 8.42 (8.45) | 8.24 (8.81) | -0.18 | -8.81 | 8.44 | 0.965 | 0.972 |
| **CD8^+^ naive** | 33.2 (25.6) | 15.6 (9.84) | -17.64 | -37.01 | 1.73 | 0.082 | 0.972 |
| **CD8^+^ terminally differentiated T-cells** | 32.6 (21.5) | 34.3 (16.2) | 1.70 | -17.33 | 20.74 | 0.852 | 0.972 |
| **CD8^+^ effector memory T-cells** | 25.8 (13.2) | 41.9 (15.2) | 16.12 | 1.88 | 30.36 | **0.029** | 0.761 |
| **Switched memory B-cells** | 16.0 (9.46) | 11.1 (7.22) | -4.89 | -13.29 | 3.52 | 0.237 | 0.972 |
| **Non-switched memory B-cells** | 7.16 (6.25) | 6.82 (7.11) | -0.34 | -7.03 | 6.35 | 0.916 | 0.972 |
| **Naïve B-cells** | 63.1 (24.2) | 68.0 (18.2) | 4.90 | -16.47 | 26.26 | 0.634 | 0.972 |
| **Double negative B-cells** | 13.7 (22.8) | 14.0 (15.1) | 0.32 | -19.01 | 19.66 | 0.972 | 0.972 |
| **NK subset 1 (CD56^-^CD16^+^)** | 45.3 (32.4) | 72.6 (13.2) | 27.30 | 2.60 | 52.01 | **0.040** | 0.972 |
| **NK subset 2 (CD56^dim^CD16^+^)** | 6.60 (6.55) | 10.2 (6.97) | 3.57 | -3.19 | 10.34 | 0.279 | 0.972 |
| **NK subset 3 (CD56^bright^CD16^-^)** | 1.26 (1.03) | 0.81 (0.43) | -0.45 | -1.24 | 0.34 | 0.252 | 0.972 |
| **Immature neutrophils** | 19.9 (23.7) | 16.9 (14.8) | -2.96 | -22.69 | 16.78 | 0.756 | 0.972 |
| **Mature neutrophils** | 77.6 (24.1) | 30.6 (16.9) | -46.98 | -67.78 | -26.17 | **<0.001** | **0.007** |

Each cell subset is shown as a percentage of the total parental cells, i.e. percentage of innate immune cells (monocytes (CD56^−^CD66b^−^CD3^−^CD19^−^ cells), NK cells (CD3^-^CD19^-^ cells), neutrophils (CD66b^+^ cells), or adaptive immune cells (CD4^+^ T-cells, CD8^+^ T-cells, and CD19^+^ B-cells), respectively. For example, each monocyte subset (classical, intermediate, or non-classical) are shown as the percentage of total monocyte populations (gated as CD56^−^CD66b^−^CD3^−^CD19^−^ cells).

LCI: lower confidence interval. UCI: upper confidence interval. Student t-test was used for comparison. P adjusted using Hochberg correction

**Supplemental Table 7. Non-parametric analysis of flow cytometry for MCI-LB and age- and sex-matched controls.**

|  | **HC** | **MCI-LB** | ***p*** | ***p-adjusted*** | **N** |
| --- | --- | --- | --- | --- | --- |
| **Classical monocytes** | 61.2 (28.0) | 53.1 (27.5) | 0.274 | 0.944 | 76 |
| **Intermediate monocytes** | 8.09 (5.86) | 8.63 (9.15) | 0.557 | 0.944 | 76 |
| **Non-classical monocytes** | 10.8 (14.3) | 13.7 (9.76) | 0.177 | 0.944 | 76 |
| **T regulatory cells** | 23.9 (20.8) | 21.4 (20.9) | 0.430 | 0.944 | 78 |
| **Non-T regulatory cells** | 67.7 (21.6) | 73.8 (22.0) | 0.104 | 0.944 | 78 |
| **Th2 cells** | 14.9 (14.1) | 14.7 (19.6) | 0.247 | 0.944 | 78 |
| **CXCR3^+^ memory B-cells** | 9.31 (8.02) | 17.7 (15.6) | 0.050 | 0.944 | 74 |
| **B regulatory (Breg) cells** | 5.46 (4.14) | 13.1 (10.7) | **0.004** | 0.113 | 74 |
| **Th1 cells** | 20.1 (10.9) | 23.2 (12.1) | 0.356 | 0.944 | 78 |
| **Th17 cells** | 6.98 (4.52) | 7.69 (12.1) | 0.142 | 0.944 | 78 |
| **Th1/Th17 cells** | 7.44 (5.16) | 7.96 (7.99) | 0.857 | 0.944 | 78 |
| **CD4^+^ central memory T-cells** | 23.1 (16.0) | 24.8 (16.3) | 0.944 | 0.944 | 78 |
| **CD4^+^ naive** | 36.2 (18.9) | 34.7 (15.3) | 0.900 | 0.944 | 78 |
| **CD4^+^ effector memory T-cells** | 30.5 (19.1) | 35.3 (9.14) | 0.247 | 0.944 | 78 |
| **CD8+ central memory T-cells** | 8.77 (10.6) | 8.24 (8.81) | 0.827 | 0.944 | 78 |
| **CD8^+^ naive** | 26.0 (21.7) | 15.6 (9.84) | 0.214 | 0.944 | 78 |
| **CD8^+^ terminally differentiated T-cells** | 32.4 (21.3) | 34.3 (16.2) | 0.719 | 0.944 | 78 |
| **CD8^+^ effector memory T-cells** | 32.8 (23.1) | 41.9 (15.2) | 0.131 | 0.944 | 78 |
| **Switched memory B-cells** | 16.1 (15.3) | 11.1 (7.22) | 0.354 | 0.944 | 74 |
| **Non-switched memory B-cells** | 8.13 (10.8) | 6.82 (7.11) | 0.901 | 0.944 | 74 |
| **Naïve B-cells** | 61.9 (23.8) | 68.0 (18.2) | 0.661 | 0.944 | 74 |
| **Double negative B-cells** | 13.8 (16.8) | 14.0 (15.1) | 0.921 | 0.944 | 74 |
| **NK subset 1 (CD56^-^CD16^+^)** | 50.3 (31.0) | 72.6 (13.2) | **0.050** | 0.944 | 76 |
| **NK subset 2 (CD56^dim^CD16^+^)** | 10.1 (13.8) | 10.2 (6.97) | 0.285 | 0.944 | 76 |
| **NK subset 3 (CD56^bright^CD16^-^)** | 1.50 (1.97) | 0.81 (0.43) | 0.891 | 0.944 | 76 |
| **Immature neutrophils** | 22.4 (24.1) | 16.9 (14.8) | 0.937 | 0.944 | 77 |
| **Mature neutrophils** | 75.3 (24.1) | 30.6 (16.9) | **<0.001** | **0.001** | 77 |

Each cell subset is shown as a percentage of the total parental cells, i.e. percentage of innate immune cells (monocytes (CD56^−^CD66b^−^CD3^−^CD19^−^ cells), NK cells (CD3^-^CD19^-^ cells), neutrophils (CD66b^+^ cells), or adaptive immune cells (CD4^+^ T-cells, CD8^+^ T-cells, and CD19^+^ B-cells), respectively. For example, each monocyte subset (classical, intermediate, or non-classical) are shown as the percentage of total monocyte populations (gated as CD56^−^CD66b^−^CD3^−^CD19^−^ cells).

Non-parametric Wilcoxon rank-sum test was used for comparison. P adjusted using Hochberg correction

**Supplemental Table 8. Linear regression analysis with three covariates (COVID infection, renal disease, and COVID vaccination) of flow cytometry measurements.**

|  | **Estimate**  **(MCI-LB vs HC)** | **95% LCI** | **95% UCI** | ***p*** | ***p-adjusted*** |
| --- | --- | --- | --- | --- | --- |
| **Classical monocytes** | -15.13 | -37.82 | 7.55 | 0.188 | 0.997 |
| **Intermediate monocytes** | -2.59 | -7.13 | 1.95 | 0.259 | 0.997 |
| **Non-classical monocytes** | 4.62 | -6.82 | 16.07 | 0.423 | 0.997 |
| **T regulatory cells** | -6.95 | -23.18 | 9.29 | 0.397 | 0.997 |
| **Non-T regulatory cells** | 11.36 | -5.46 | 28.19 | 0.183 | 0.997 |
| **Th2 cells** | 4.92 | -7.07 | 16.90 | 0.416 | 0.997 |
| **CXCR3^+^ memory B-cells** | 8.31 | 1.20 | 15.41 | **0.023** | 0.566 |
| **B regulatory (Breg) cells** | 11.05 | 7.02 | 15.09 | **<0.001** | **<0.001** |
| **Th1 cells** | 1.02 | -8.09 | 10.12 | 0.824 | 0.997 |
| **Th17 cells** | 3.84 | -0.72 | 8.39 | 0.097 | 0.997 |
| **Th1/Th17 cells** | 1.30 | -3.26 | 5.85 | 0.572 | 0.997 |
| **CD4^+^ central memory T-cells** | 4.29 | -8.71 | 17.29 | 0.513 | 0.997 |
| **CD4^+^ naive** | -0.03 | -15.25 | 15.18 | 0.997 | 0.997 |
| **CD4^+^ effector memory T-cells** | 0.30 | -14.48 | 15.09 | 0.967 | 0.997 |
| **CD8+ central memory T-cells** | 0.51 | -7.97 | 8.99 | 0.904 | 0.997 |
| **CD8^+^ naive** | -4.17 | -19.15 | 10.81 | 0.581 | 0.997 |
| **CD8^+^ terminally differentiated T-cells** | 4.36 | -12.41 | 21.13 | 0.606 | 0.997 |
| **CD8^+^ effector memory T-cells** | -0.71 | -17.51 | 16.09 | 0.933 | 0.997 |
| **Switched memory B-cells** | -0.79 | -12.59 | 11.01 | 0.894 | 0.997 |
| **Non-switched memory B-cells** | -2.03 | -10.54 | 6.49 | 0.637 | 0.997 |
| **Naïve B-cells** | 5.92 | -13.16 | 24.99 | 0.538 | 0.997 |
| **Double negative B-cells** | -3.10 | -16.26 | 10.05 | 0.639 | 0.997 |
| **NK subset 1 (CD56^-^CD16^+^)** | 10.84 | -9.68 | 31.35 | 0.296 | 0.997 |
| **NK subset 2 (CD56^dim^CD16^+^)** | -1.29 | -12.19 | 9.62 | 0.815 | 0.997 |
| **NK subset 3 (CD56^bright^CD16^-^)** | -0.13 | -1.60 | 1.33 | 0.856 | 0.997 |
| **Immature neutrophils** | -14.14 | -31.40 | 3.13 | 0.107 | 0.997 |
| **Mature neutrophils** | -33.04 | -50.35 | -15.74 | **<0.001** | **0.008** |

Each cell subset is shown as a percentage of the total parental cells, i.e. percentage of innate immune cells (monocytes (CD56^−^CD66b^−^CD3^−^CD19^−^ cells), NK cells (CD3^-^CD19^-^ cells), neutrophils (CD66b^+^ cells), or adaptive immune cells (CD4^+^ T-cells, CD8^+^ T-cells, and CD19^+^ B-cells), respectively. For example, each monocyte subset (classical, intermediate, or non-classical) are shown as the percentage of total monocyte populations (gated as CD56^−^CD66b^−^CD3^−^CD19^−^ cells).

LCI: lower confidence interval. UCI: upper confidence interval. P adjusted using Hochberg correction
